# Chemical Epigenetic Regulation Secondary Metabolites Derived from *Aspergillus sydowii* DL1045 with Inhibitory Activities for Protein Tyrosine Phosphatases

**DOI:** 10.3390/molecules29030670

**Published:** 2024-01-31

**Authors:** Xuan Shi, Xia Li, Xiaoshi He, Danyang Zhang, Chunshan Quan, Zhilong Xiu, Yuesheng Dong

**Affiliations:** 1 School of Bioengineering, Dalian University of Technology, Dalian 116024, China; xuan80904@mail.dlut.edu.cn (X.S.); lix070128@163.com (X.L.); syingjjip@163.com (X.H.); zhangdanyang96@mail.dlut.edu.cn (D.Z.); zhlxiu@dlut.edu.cn (Z.X.); 2 College of Life Science, Dalian Minzu University, Dalian 116600, China; mikyeken@dlnu.edu.cn

**Keywords:** chemical epigenetic regulation (CER), protein tyrosine phosphatases (PTPs), *Aspergillus sydowii*, secondary metabolites (SMs)

## Abstract

Protein tyrosine phosphatases (PTPs) are ubiquitous in living organisms and are promising drug targets for cancer, diabetes/obesity, and autoimmune disorders. In this study, a histone deacetylase inhibitor called suberoylanilide hydroxamic acid (SAHA) was added to a culture of marine fungi (*Aspergillus sydowii* DL1045) to identify potential drug candidates related to PTP inhibition. Then, the profile of the induced metabolites was characterized using an integrated metabolomics strategy. In total, 46% of the total SMs were regulated secondary metabolites (SMs), among which 20 newly biosynthesized metabolites (10% of the total SMs) were identified only in chemical epigenetic regulation (CER) broth. One was identified as a novel compound, and fourteen compounds were identified from *Aspergillus sydowii* first. SAHA derivatives were also biotransformed by *A. sydowii* DL1045, and five of these derivatives were identified. Based on the bioassay, some of the newly synthesized metabolites exhibited inhibitory effects on PTPs. The novel compound sydowimide A (A11) inhibited Src homology region 2 domain-containing phosphatase-1 (SHP1), T-cell protein tyrosine phosphatase (TCPTP) and leukocyte common antigen (CD45), with IC_50_ values of 1.5, 2.4 and 18.83 μM, respectively. Diorcinol (A3) displayed the strongest inhibitory effect on SHP1, with an IC_50_ value of 0.96 μM. The structure–activity relationship analysis and docking studies of A3 analogs indicated that the substitution of the carboxyl group reduced the activity of A3. Research has demonstrated that CER positively impacts changes in the secondary metabolic patterns of *A. sydowii* DL1045. The compounds produced through this approach will provide valuable insights for the creation and advancement of novel drug candidates related to PTP inhibition.

## 1. Introduction

Microbial secondary metabolites (SMs) are a significant source of leading pharmaceutical compounds. *Aspergillus*, a filamentous fungus widely distributed in nature, is rich in enzymes and produces a vast number of bioactive SMs, including polyketides, nonribosomal peptides, terpenoids and numerous hybrids of these classes [1,2]. *Aspergillus sydowii*, also known as *Emericella sydowii*, is mainly isolated from marine sources and produces unique and biologically active SMs [3]. To date, 4361 natural products have been identified from the fungi of the genus *Aspergillus*, 60 of which are derived from *A. sydowii* based on the Reaxys database (https://www.reaxys.com/). For example, three diketopiperazine alkaloids (6-methoxyspirotryprostatin B, 18-oxotryprostatin A, and 14-hydroxyterezine D) were isolated from *A. sydowii* and exhibited cytotoxicity against A-549 cells, with IC_50_ values of 8.29, 1.28, and 7.31 μM, respectively [4]. The compounds aspergillusene A, diorcinol and (*Z*)-5-(Hydroxymethyl)-2-(6′) methylhept-2′-en-2′-yl)-phenol showed modest antimicrobial activity against *K. pneumonia*, with MIC values of 21.4, 21.7 and 10.7 mM, respectively [5]. Nonetheless, the entire genome of *A. sydowii* uncovered numerous genes for biosynthesis that remained undetected under standard conditions [6]. Therefore, the potential biosynthetic ability of *A. sydowii* should be further explored.

SM clusters in *Aspergillus* species are typically located near the telomeres of chromosomes, and their expression is related to the epigenetic modification of histones [7]. Histone structural modifications, such as acetylation and methylation, affect chromatin structure. Acetylated histone tails are generally associated with an open state, whereas deacetylated histones are linked to closed chromatin [8]. Therefore, inhibiting enzymes that catalyze deacetylation and DNA methyltransferases may reveal the gene loci of undiscovered SMs and produce new natural products. In fact, chemical epigenetic regulation (CER)—the addition of histone deacetylase (HDAC) inhibitors and DNA-methyltransferase (DNMT) inhibitors to culture media—has been reported to be useful for producing new SMs from many *Aspergillus* strains [9,10,11,12]. Our group discovered that *Aspergillus aculeatus* DL1011 was induced by the HDAC inhibitor hydroxamic acid (SBHA), leading to the generation of new compounds. Thirteen newly induced SMs were identified via a combination of tools (MetaboAnalyst, MS-DIAL, SIRIUS and GNPS) [13]. Moreover, an analogous comprehensive metabolomics approach was used to investigate the collective alterations in the microbial metabolites profile of *A. sydowii* and *Bacillus subtilis* co-culture and to clarify the structural details of 25 compounds [14]. The combination of these metabolomic tools enabled a comprehensive understanding of a comprehensive sample and allowed for the rapid identification of targets of interest in *Aspergillus*. Nevertheless, the accuracy of this method must be further tested.

PTPs are a class of metal-free enzymes that are widely distributed in organisms and play crucial roles in human diseases, such as cancer, diabetes/obesity, and autoimmune dysfunctions. Therefore, PTPs have emerged as potential new drug targets [15]. Some PTPs, such as protein tyrosine phosphatase-1B (PTP1B), T-cell protein tyrosine phosphatase (TCPTP) and leukocyte common antigen (CD45), impair insulin activity on glucose metabolism by dephosphorylating insulin receptors [16,17]. The protein tyrosine phosphatase Src homology region 2 domain-containing phosphatase-1 (SHP-1) can suppress activation-promoting signaling pathways in hematopoietic cells and regulate glucose homeostasis by modulating insulin signaling in the liver and muscle as well as hepatic insulin clearance [18]. Consequently, the development of effective and specific inhibitors for PTPs has attracted considerable attention in recent years.

In this study, an integrated metabolomics strategy that was previously established—which included MetaboAnalyst, MS-DIAL, SIRIUS and GNPS—was applied to describe alterations in SMs within *A. sydowii* DL1045 by CER. Several of the newly induced metabolites were purified and identified via NMR spectroscopy, which verified the precision of the metabolomics approach. The biological activities of the newly synthesized compounds and their effects on protein tyrosine phosphatases (PTPs) were analyzed, and the underlying mechanism was investigated using the molecular docking technique.

## 2. Results

### 2.1. Effects of CER on Growth of A. sydowii DL1045

**Figure 1:**
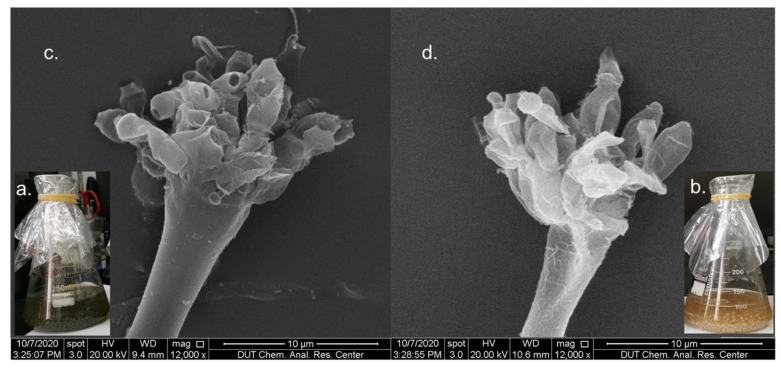
Fermentation broth and mycelial morphology of *A. sydowii* DL1045 cultivated with 250 μM SAHA (**b**,**d**) and in control culture (**a**,**c**) on 10 days.

After *A. sydowii* DL1045 in rice extract culture medium was treated with three different histone deacetylase inhibitors, including suberoylanilide hydroxamic acid (SAHA), hydroxamic acid (SBHA) and nicotinamide, the fermentation status of the fungi was observed. The results showed that the color of the fermentation broth in the SAHA group was significantly different from that in the control group. The fermentation broth of *A. sydowii* DL1045 in the control group was dark green, whereas the fermentation broth in the 250 μM SAHA treatment group was golden yellow (Figure 1a,b). In addition, after 10 days, the weight of metabolites was 3.5 times lower in the SAHA group than in the control group (Figure S1, Supplementary Materials). This phenomenon indicated that SAHA may slow the growth of *A. sydowii* DL1045. This conjecture was confirmed by the results of scanning electron microscopy, and after 10 days of fermentation, the spores detached from the sporangium after maturation in the later stage of fermentation (Figure 1c). However, the addition of SAHA inhibited mycelial growth and spore germination (Figure 1d). These results indicated that changes in the growth of *A. sydowii* DL1045 were induced by CER.

### 2.2. Metabolites Profile Induced by Adding SAHA into A. sydowii DL1045

**Figure 2:**
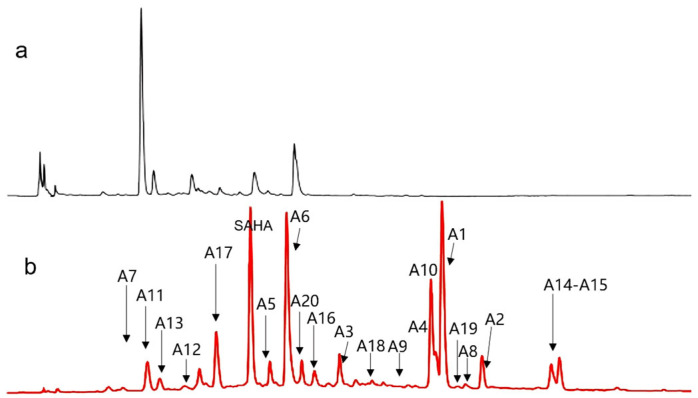
HPLC profile for the extracts from *A. sydowii* DL1045 grown in the presence of (**b**) 250 μM SAHA and (**a**) in control culture was analyzed based on UV absorption at 254 nm.

Subsequent research delved into the influence of various SAHA concentrations on metabolites by adding five different concentrations (50, 100, 150, 250, and 500 μM) to the fermentation broth of *A. sydowii* DL1045. It was observed that a concentration of 250 µM SAHA prompted the most pronounced alterations in the metabolite spectrum profile (Figure 2 and Figure S2). To further explore the influence of SAHA on the composition of SMs, a detailed study was conducted using the metabolic analysis process previously established. The EtOAc extracts of *A. sydowii* DL1045 that were treated and not treated with 250 μM SAHA were examined using LC-MS/MS. After the features of low abundance and blanks were discarded using MS-DIAL software (version 4.9) and a minimum peak height threshold was set, a total of 200 features were identified.

**Figure 3:**
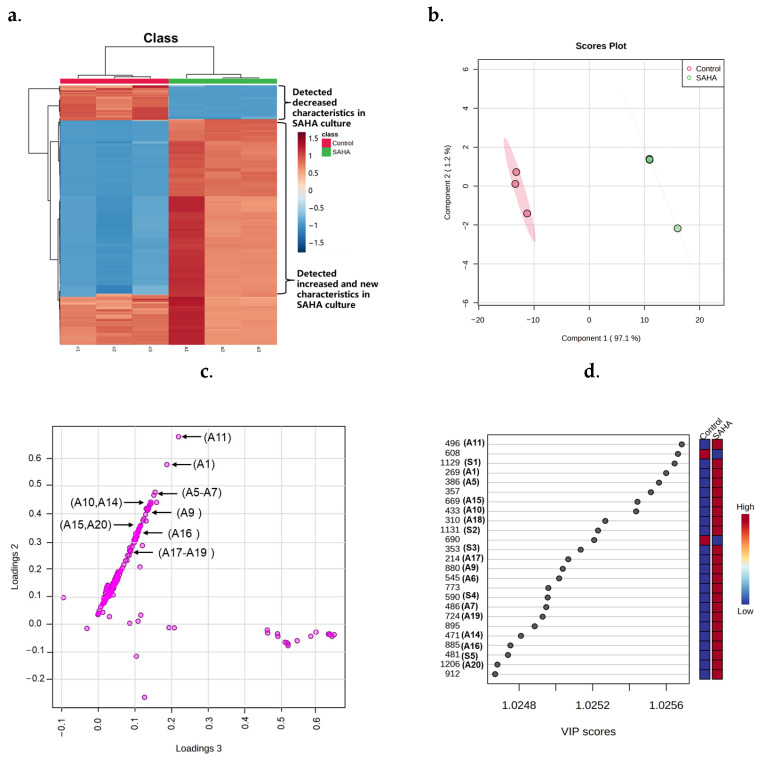
The heatmap and PLS-DA of metabolomics of control and 250 μM suberoylanilide hydroxamic acid (SAHA)-regulated *A. sydowii* DL1045. (**a**) Hierarchical clustering analysis (HCA) was conducted on the 200 most variably significant features depicted on a heatmap with a gradient from red (high abundance) to blue (low abundance). (**b**) PLS-DA scores plot results based on the LC-MS data obtained for groups of SAHA and control. (**c**) PLS-DA loading plot of all metabolite features. (**d**) PLS-DA VIP score of the top features. The compound numbers in the brackets are newly bio-synthetized metabolites identified in this study.

MetaboAnalyst was used to examine several features, as summarized in Figure 3. A heatmap of 200 characteristics obtained via hierarchical clustering analysis (HCA) demonstrated that CER caused extensive alterations in the metabolome (Figure 3a). A heatmap showed that 15% (30/200) and 21% (42/200) of the characteristics increased and decreased, respectively, in abundance following SAHA treatment (Table S1). Furthermore, 20 compounds were exclusively found in SAHA-treated cultures, showing that 10% of the SMs were created for the first time by CER. As a result, the regulated SMs occupied 46% of the total SMs, suggesting a substantial change in the SMs profile caused by adding SAHA to the broth. Partial least squares discriminant analysis (PLS-DA) of these features revealed substantial separation between the experimental groups and the control group. In the score plot, the clusters representing the CER-treated samples were markedly segregated from those of the untreated cultures, highlighting that the composition of metabolites was significantly altered (Figure 3b). On the PLS-DA loading plot, the 20 newly induced compounds were positioned distant from the origin, which was a significant factor leading to the differences among the experimental groups. In contrast, the attributes with minimal influence on group differentiation were located closer to the origin (Figure 3c). As observed with the previous phenomenon [13], the 20 compounds exhibited a strong linear association. Moreover, the variable importance in projection (VIP) score data obtained via PLS-DA suggested that newly induced compounds (A1, A5–A7, A9–A11, A14–A20 and S1–S5) were the top contributors (Figure 3d). These results revealed that the newly biosynthesized compounds developed from CER play a substantial role in group classification.

**Figure 4:**
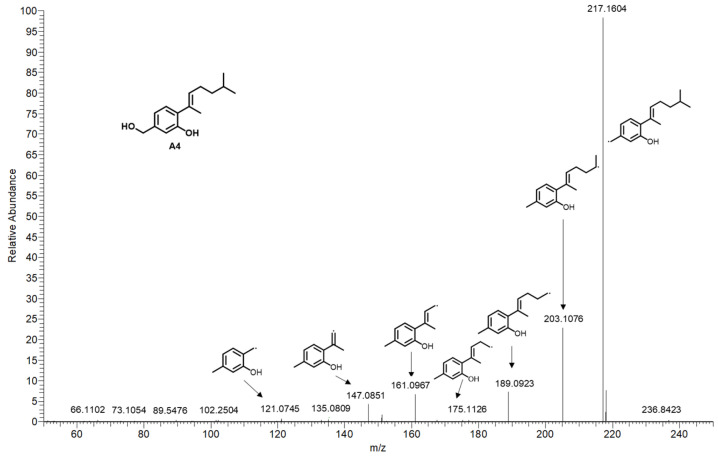
MS/MS spectrum of A4 (*m*/*z* 235.1692) in positive mode.

To determine the structures of the SMs that were emphasized as key features by the VIP score, an integrated approach was utilized to identify 20 features (Figure 2). In this method, the following levels of structure annotation are integrated: level 1, which is based on MS/MS databases linked to MS-DIAL; level 2, which utilizes CSI: FingerID in SIRIUS; level 3, which consists of structure annotation supported by the global natural product social molecular network (GNPS); and level 4, which confirms the structures through isolation and purification, together with NMR spectrum analysis, as previously established [13]. For example, A4 was not matched in MSP spectral databases (level 1), so level 2 identification was required. A4 was detected as *m*/*z* 235.1692 [M+H]^+^, and the molecular formula was established as C_15_H_22_O_2_ due to the SIRIUS score of 100% among the candidate formulas. The fragmentation tree of A4 revealed that the prominent fragment at *m*/*z* 217.1586 resulted from the parent molecular ion at *m*/*z* 235.1692 via the loss of H_2_O (18 Da). Subsequent fragmentation led to a fragment at *m*/*z* 203.1076 through the loss of CH_2_ (14 Da) from *m*/*z* 217.1586. This pattern continued with the formation of fragments at *m*/*z* 189.0923, 175.1126, 161.0967, and 147.0851, each through the successive loss of CH_2_ (14 Da) from the preceding fragment. Ultimately, a fragment at *m*/*z* 121.0745 was produced via the loss of CH_2_ (14 Da) from the fragment at *m*/*z* 147.0851 (Figure 4). After evaluating the molecular fingerprint that was predicted through CSI: FingerID, A4 was identified as aspergillusene A, which had the highest match score (65.1%) against the Natural Products and PubChem databases. Thus, six newly induced features (A5, A10, A15–A17, and A20) were identified by level 1 in our strategy. Another 13 unmatched features (A1–A4, A6–A9, A12–14, A18, and A19) were annotated by level 2 using the integration of CSI: FingerID in SIRIUS (Table 1). For instance, the molecular formula of compound A1 was C_15_H_20_O, which was indicated by the HRESIMS *m*/*z* 217.1586 [M+H]^+^ (calcd: *m*/*z* 217.1592). It was identified as (−)-1,2-dehydro-α-cyperone, a stress compound in TMV-infected leaves of *Nicotiana undulata* [19]. The molecular formulas of compounds A12 and A14 were C_17_H_14_O_5_ and C_16_H_14_O_7_, which were indicated by the HRESIMS *m*/*z* 299.0903 [M+H]^+^ (calcd: *m*/*z* 299.0919) and *m*/*z* 319.0813 [M+H]^+^ (calcd: *m*/*z* 319.0818), respectively. These compounds were identified as emodin-1,6-dimethyl ether and lecanoric acid, respectively. These compounds were isolated from *Aspergillus fumigatus* [20] and *Aspergillus versicolor* [21], respectively. Among the thirteen compounds in Level 2, eight (A1, A6–A7, A9, A12, A14, A18, and A19) were identified from *A. sydowii* for the first time.

After levels 1 and 2, most newly synthesized metabolites were identified successfully except for feature A11, so this feature was forwarded to level 3, which can cluster similar structures and analogs from various samples via LC-MS/MS and is unaffected by variations in retention time [36]. According to the GNPS analysis, feature A11 did not express any cluster with the other identified compounds; thus, this feature was classified as level 4 and was identified mainly via isolation, purification, and NMR analysis. Compound A11 was obtained as a yellow oily solid using a sequential chromatographic process involving a silica gel column, Sephadex LH-20 column, middle-pressure ODS column, and preparative HPLC. The compound A11 had a molecular formula of C_15_H_16_O_4_N_2_ with nine degrees of unsaturation on the basis of positive ESIHRMS *m*/*z* 289.1185 [M+H]^+^ and 287.1034 [M-H]^−^ (Figure S3). The UV absorption of the compound was set at 220 and 309 nm. The IR spectrum showed the presence of an amino group (3200 cm^−1^), methylene (2924 cm^−1^), amide (1657 cm^−1^), benzene ring (1503 cm^−1^), methyl (1345 cm^−1^), and methoxy (1268 cm^−1^) (Figure S4). However, A11 was obtained as an inseparable mixture of isomers in a ratio of 2:1 (A11a and A11b), as deduced from the paired signal peaks in the ^1^H and ^13^C spectra. The structure of the major isomer (A11a) was deduced via 1D NMR resonance. The 1D NMR data of A11a were categorized into two methyl groups (including a methoxy), one methylene, six sp^2^ methines, one sp^3^ methine, and five nonprotonated carbons, including an α/β-unsaturated ketone carbon; this result indicated that A11a contained one additional ring to match the degree of unsaturation (Table 2, Figures S5 and S6).

**Figure 5:**
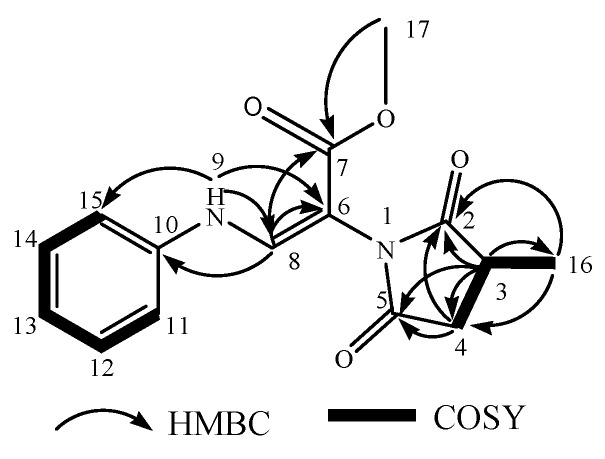
The key COSY, HMBC of A11a.

According to the 2D NMR data, the presence of a phenyl group was confirmed via key COSY correlations from H-11 through H-15 and HMBC correlations from H-9 (NH) to C-6, C-8, C-11, and C-15, which also revealed its attachment to the 9-N atom. The COSY correlations of H-3, H2-4 and H3-15 and HMBC correlations of H-3, H2-4 and H3-15 with C-2(δ180.05) and C5 (δ175.98) led to the identification of a 3-methylpyrrolidine-2,5-dione [37]. Thus, the planar structure of A11a was determined (Figure 5 and Figures S7–S9).

**Figure 6:**
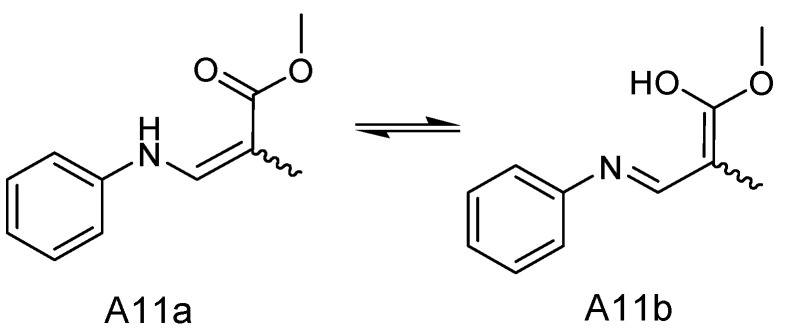
The interconverting geometric isomers of sydowimide A (A11).

A comparison of the NMR data of A11a and A11b showed that the compounds contained similar planar core structures. The primary significant differences were the chemical shifts at carbon positions C-2, C-3 and C-16, as shown in Table 2. This suggested that A11 may have existed as a set of interconverting geometric isomers in solution, as depicted in Figure 6. Due to the presence of ketoenamine in its structure, A11 can easily form tautomerize with enol-imine in solution and reach a stable state. This finding was consistent with previously reported results of the tautomerism of acetylacetone and various diamine condensation products [38]. These bases were reported to exist in the ketamine form, which was the same as the main existing form of the A11a. Thus, the structure of A11b was also elucidated (Figure 6).

**Figure 7:**
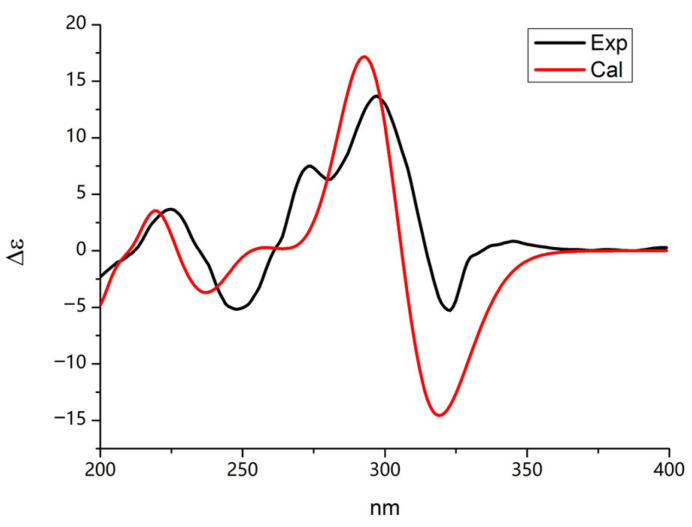
Experimental and calculated electronic circular dichroism (ECD) spectra of sydowimide A (A11) (black, experimental in MeOH; red, calculated in MeOH).

For A11a and A11b, the only stereochemical configuration was at C-3. To prove the absolute configuration, the absolute configuration of C-3 of A11 was calculated as the *R* configuration by using Gaussian 09 in the ECD spectra (Figure S10). The conformers were optimized through DFT at the B3LYP/6-31G (d) level within a methanol environment. Energy calculations were performed using TDDFT at the B3LYP/6-31G (d, p) level in MeOH, incorporating the PCM model (Tables S2 and S3). The calculated CD spectrum of A11, which was calculated as the *R* configuration, corresponded closely to the experimental CD data, as shown in Figure 7. Therefore, compound A11 was identified as a novel compound and named sydowimide A, which contained two isomers, 2-(3*R*-methyl-2,5-dioxo-pyrrolidin-1-yl)-3-phenylamino-acrylic acid methyl ester (sydowimide A11a) and (1-hydroxy-1-methoxy-3-(phenylimino) prop-1-en-2-yl)-3*R*-methylpyrrolidine-2,5-dione (sydowimide A11b).

**Figure 8:**
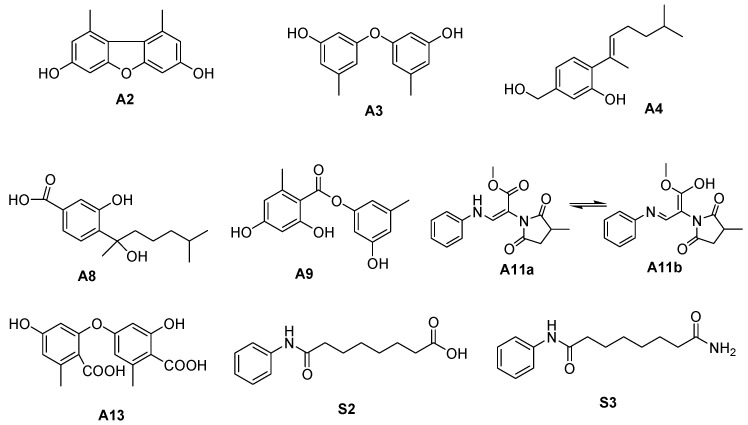
Structures of the isolated metabolites for structural identification.

Consequently, all 20 features depicted in Figure S11, which were induced by CER, were successfully identified through our integrated approach in which a comprehensive software platform, computational methods and experiments were combined. Among these, fifteen compounds, which included one novel compound and fourteen previously known compounds, were detected for the first time in *A. sydowii*, representing 75% of the newly biosynthesized metabolites. To confirm the reliability of our strategy, seven new biosynthesized metabolites (A2–A4, A8–A9, A11 and A13) with substantial quantities were further isolated and purified through chromatography using a silica gel column, Sephadex LH-20 column, middle-pressure ODS column, and preparative HPLC, achieving over 95% purity. Their structures were identified through NMR analysis (as shown in Figure 8, with detailed compound information provided in the Supplementary Material). The NMR data for the isolated compounds corroborated the structures predicted through our approach, further validating the precision of our strategy.

### 2.3. Identification of SAHA Biotransformation Products

**Figure 9:**
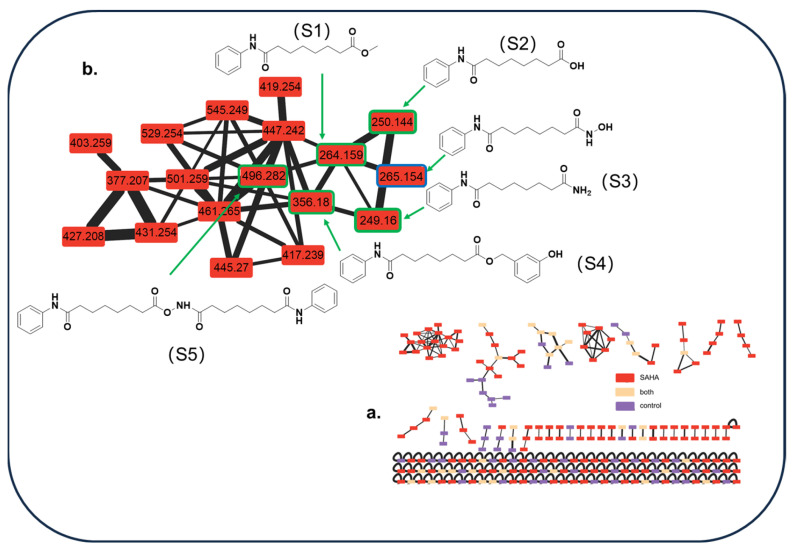
Global natural product social molecular network (GNPS) enabled the discovery of suberoylanilide hydroxamic acid (SAHA) derivatives. (**a**). Molecular network of compounds in different cultures (red in SAHA culture, purple in control culture and yellow in both cultures); (**b**). a cluster including 5 SAHA derivatives (*m*/*z* 249.160, 250.144, 264.159, 356.180, 496.282).

In level 3 (GNPS) of our strategy, a complex molecular cluster containing 18 nodes that included the regulator SAHA (*m*/*z* 265.154) was not related to the metabolites of *A. sydowii* DL1045 and was present in only the SAHA-treated cultures (red nodes) (Figure 9a). This result indicated that a series of large SAHA derivatives were produced. Based on MS/MS data and CSI: FingerID in SIRIUS, five SAHA derivatives were identified as methyl 8-oxo-8-(phenylamino) octanoate (S1), suberanilic acid (S2), 3-hydroxybenzyl 8-oxo-8-(phenylamino)octanoate (S3), octanedioic acid phenylamide (7-phenylcarbamoyl-heptanoyloxy) amide, respectively, (S4) and octanedioic acid phenylamide (7-phenylcarbamoyl-heptanoyloxy)amide (S5), and their structures are shown in Figure 9b. Compounds S2 and S3 with high content in the fermentation broth were also isolated and purified to give structural information through NMR analysis. The fermentation broth without *A. sydowii* and the fermentation broth after 10 days with *A. sydowii* showed that the amount of SAHA was reduced by 11% (Figure S12), indicating that SAHA was partly converted into its derivatives and that the CER effects were generated by SAHA and its biotransformation products.

### 2.4. Biological Activity Assay

The five isolated newly biosynthesized metabolites (A3, A4, A9, A11 and A13) were also evaluated for their inhibitory activity against several PTPs, including PTP1B, TCPTP, SHP1 and CD45 (Table 3), and anti-nematode activity (Table S4). For PTPs, most compounds exhibited potent activity against SHP1, of which diorcinol (A3) showed the highest activity, with an IC_50_ value of 0.96 μM. The novel compound A11 showed relatively strong inhibitory activities with an IC_50_ of 1.50 μM against this enzyme. Most of the tested compounds, except for aspergillusene A (A4), showed CD45 inhibitory activity. However, aspergillusene A (A4) and lecanorin (A9) showed potent activity against PTP1B, with IC_50_ values of 9.65 μM and 7.60 μM, respectively. Among the compounds, diterpenes diorcinol (A3) and diorcinol acid (A13) showed inhibition activities against SHP1 and CD45. In addition, only the new compound sydowimide A (A11) displayed inhibition activities against TCPTP with an IC_50_ value of 2.4 μM. For anti-nematode activity, only diorcinol (A3) exhibited activity, and the IC_50_ value was 50 μM.

**Figure 10:**
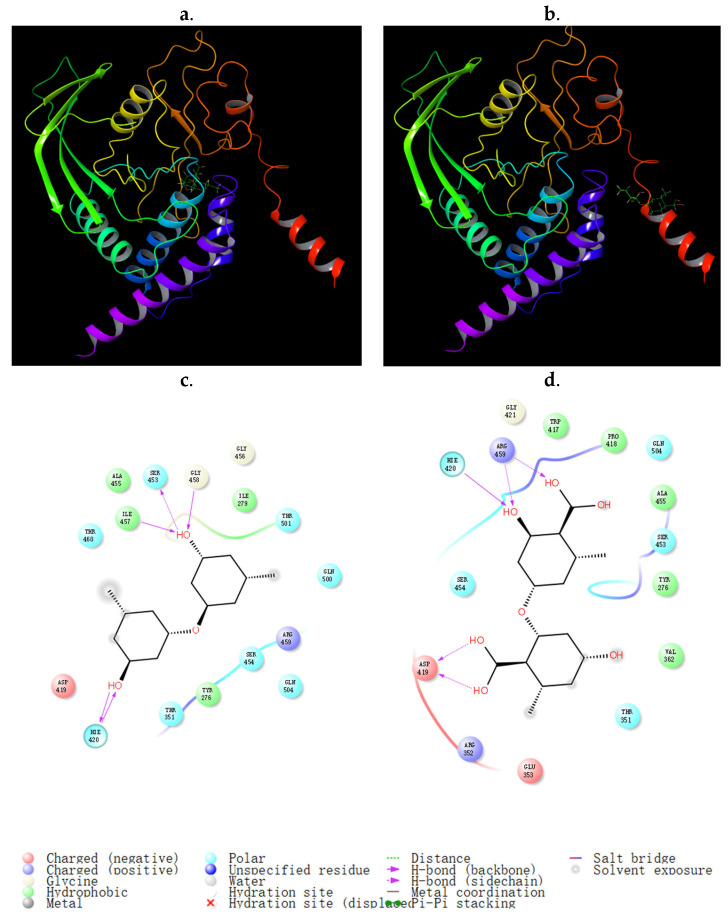
Representation of the binding mode of compounds A3 (**a**,**c**) and A13 (**b**,**d**) in the active site and the inactive site of Src homology region 2 domain-containing phosphatase-1 (SHP1).

In the SHP1 assay, different activity was observed for two analogs, A3 and its carboxylic acid (A13), suggesting that the substitution of the carboxyl group harmed the inhibitory activity of SHP1. To verify the relationship between the structure and activity of the proteins, a docking study was performed using Schrodinger software (maestro version 10.2) (Figure 10). The binding glide scores of A3 and A13 to SHP1 predicted that A3 and A13 were bound to the active site and the inactive site [39], respectively (Table 4). The docking results showed that the docked pose of A3 with SHP1 had a docking glide score of −5.705 kcal/mol at site 2 (active site) (Figure 10a), and the major interactions between A3 and SHP1 were hydrogen bonds between Hie 420, Ile 457, Ser 453, and Gly 458 and the hydroxyl groups and hydrophobic interactions (which were composed of Ile 279, Ile 457, Ala 455 and Tyr 276) (Figure 10c). The docked pose of A13 with SHP1 had a docking glide score of -4.832 kcal/mol at site 3 (Figure 10b), and the major interactions between A13 and SHP1 were hydrogen bonds between Gln 252, Glu 265, Gln 250, and Gln 254 (Figure 10d). Therefore, A13 was more likely to bind to the inactive site of SHP1 because of steric hindrance of the carboxyl groups. Compared to A13, compound A3 possessed a higher docking glide score and stronger interactions with the SHP1 active site, which explained its greater activity.

Since the new compound A11 showed inhibitory activity against SHP1, the molecular docking examination of the keto and enol isomers of compound A11 was also performed (Table S5 and Figure S13). The results showed that A11a, as the main form of interconversion, was easily bound to the active site of SHP1 at site 2, with a docking glide score of −4.054 kcal/mol. In addition, the major interactions between A11a and SHP1 were hydrogen bonds between Arg 459, Gly 421 and Gly 504 and the hydroxyl groups and hydrophobic interactions (which were composed of Ala 455, Ttr 276, Ile 457, Ile 279, Val 422, Ala 503, Trp 417 and Pro 418). Furthermore, A11b tended to bind to the inactive site due to the presence of the enol structure. Therefore, it can be inferred that the inhibitory activity of A11 against SHP1 may result from the two tautomers binding to the active site and the inactive site, respectively.

To further evaluate the possibility of bioactive compounds becoming potential drugs, the physicochemical parameters, pharmacokinetic properties and absorption, distribution, metabolism and excretion (ADME) parameters, and drug-likeness rule scores of the compounds were predicted using the online program SwissADME [40] (Table S6). These five compounds (A3, A4, A9, A11 and A13) have molecular weights ranging from 230 to 320 and consensus Log P values between 1.44 and 3.56, indicating high lipophilicity. The log S calculations showed that the compounds were soluble in water, with A11 being highly soluble and the others being moderately soluble. This suggests that these compounds can be well absorbed. All the compounds exhibited high gastrointestinal (GI) absorption, but only A3 and A4 displayed good blood–brain barrier permeability (BBB permeant). The bioavailability score was 55%, indicating that there was a 55% chance of having at least 10% bioavailability. Moreover, these five compounds met the five different drug-likeness criteria of Lipinski, Ghose, Veber, Egan, and Muegge and demonstrated good drug-like properties.

## 3. Discussion

*A. sydowii* is a fungal species with wide applications, especially in the pharmaceutical field. The fungi produce bioactive SMs with chemical and pharmaceutical properties, such as heterospirocyclic γ-lactam derivatives [41], sesquiterpenes and xanthones [42], diketopiperazine dimer [43] as well as enzymes of industrial interest, such as lipases, α-amylases, and xylanases [3]. To explore the biosynthetic potential of *A. sydowii* further, various strategies have been applied to activate the silent metabolic pathways. The utilization of the OSMAC method has been demonstrated to effectively induce *A. sydowii* to synthesize acetylcholinesterase inhibitors [44]. The secondary metabolites in the co-culture of *A. sydowii* EN-534 and *P. citrinum* EN-535 were investigated, leading to the discovery of two novel derivatives of citrinin, as well as eight previously identified derivatives [45].

Our group found that the co-culture of *A. sydowii* and *Bacillus subtilis* induced the production of antibacterial compounds [14,46]. A previous study showed that adding the DNA methyltransferase inhibitor, 5-azacytidine, to *A. sydowii* culture broth resulted in the discovery of the following new bisabolane-type sesquiterpenoids: (7*S*,11*S*)-(+)-12-hydroxysydonic acid, (7*S*)-(+)-7-*O*-methylsydonol and 7-deoxy-7,14-didehydrosydonol [47]. In this study, we added SAHA, a common small-molecule histone deacetylase inhibitor, to *A. sydowii* culture broth and induced the production of a variety of active compounds. For example, the compound A6 (methyl trimethoxycinnamate) showed inhibitory activity against *Staphylococcus aureus* [25]. Compound A7 (radicamine A) has been reported to exhibit α-glucosidase inhibitory activity [26]. Compound A9, known as lecanorin, markedly inhibited the radicle growth and germination of the two weeds [27]. Furthermore, a novel compound, sydowimide A (A11), was identified within the *A. sydowii* culture following treatment with SAHA. As far as we know, SMs containing 2,5-dioxo-pyrrolidin structures have not been found in *A. sydowii*, indicating that the biosynthetic potential of *A. sydowii* warrants further exploration.

Epigenetic strategies have been instrumental in elucidating the regulation of SM formation in fungi. The histone acetylation level is a key factor that affects the genomic expression of SMs. A previous study showed that deleting *hosA*, which is the classical HDAC gene in *Aspergillus nidulans*, not only altered morphological changes and pigment production but also had opposite effects on the biosynthesis of penicillin and orsenic acid [48]. The deletion of another HDAC gene, *hdaA,* from *A. nidulans* activated two telomere-proximal gene clusters, leading to an increase in the production of antibiotics and corresponding toxins; however, telomere-distal gene clusters were not activated [49]. Moreover, the metabolome of an *A. nidulans rpdA*(Class I-Rpd3 HDACs) knockdown mutant resembled that of the treatment of SAHA, inducing the upregulation and downregulation of several SMs [50,51]. However, histone gene modification studies have been limited to model fungi with clear genomic information. Therefore, in the present study, the common small-molecule histone deacetylase inhibitor SAHA was added to the broth of *A. sydowii* DL1045 with vague genomic information. The significant change in the profile suggested that some gene clusters associated with SM synthesis were activated, which provided a theoretical basis for the subsequent study of silent genes activated by *A.sydowii*.

In addition, our data indicated that SAHA was partially biotransformed into derivatives during the fermentation of *A. sydowii*. This phenomenon is still rare during CER studies, as it has been predicted via GNPS only for *Penicillium* sp. [52] and endophytic fungus *Botryosphaeria mamane* [53]. However, no previous reports have documented the conversion rate of SAHA or purified its derivatives to verify the prediction accuracy. To the best of our knowledge, this was the first report on the biotransformation of SAHA by a fungus from the genus of *Aspergillus*. In our study, GNPS prediction and structural identification data indicated that *A. sydowii* had a potent effect on the biotransformation of SAHA, with up to 18 nodes detected via GNPS and a conversion rate of 11%. Moreover, some SAHA analogs, such as dehydroxylated SAHA (S3), were reported for the first time as biotransformation products. Our results demonstrated that the biotransformation of SAHA by *A. sydowii* might be an alternative means to obtain SAHA analogs. Of course, further work is still needed to identify the biotransformation products predicted via GNPS and to optimize the biotransformation conditions to increase the conversion rate.

On the other hand, derivatives of SAHA have been reported to be derived through chemical synthesis, and compounds S1 and S2 displayed HDAC inhibitory activity [54,55]. Thus, it was plausible to infer that the epigenetic alterations observed in HDAC inhibitor-treated *A. sydowii* cultures could be attributed to the combination of SAHA derivates rather than SAHA alone. A prior study demonstrated that the addition of both SBHA and RG-108, DNA methyltransferase inhibitors resulted in the production of polyketide with a novel skeletal structure from the medium of *Isaria tenuipes* [56]. In this study, 75% of the newly biosynthesized metabolites were first identified in *A. sydowii*, but the efficacy of the HDAC inhibitor mixture in activating the silent genes of SMs required further investigation.

In this study, five isolated newly induced compounds (A3, A4, A9, A11 and A13) exhibited significant activities against PTPs, including PTP1B, TCPTP, SHP1 and CD45. The novel compound sydowimide A (A11) displayed inhibitory activities against SHP1, TCPTP and CD45 and showed the strongest inhibitory activity against SHP1. Compared with the docking results, A3 and A11 tended to bind to the active site, but the docking score of A11 was lower, indicating that the interaction between A11 and SHP1 was weaker than that between A3 and SHP1, which was also consistent with the experimental results. The SwissADME analysis results indicated that the metabolites of *A. sydowii* DL1045 could be developed as medicine and could be the basis of subsequent studies in vitro and in vivo. However, further research is needed to elucidate the structure-activity relationships of these compounds, which will contribute to the development of innovative treatments for diseases such as immune disorders, diabetes and cancer.

## 4. Materials and Methods

### 4.1. Fungal Material

*A. sydowii* DL1045 was retrieved from deep-sea mud sediment off the coast of Dalian, Liaoning Province, 121.55 °E and 38.88 °N, China. *A. sydowii* DL1045 was cultivated on potato dextrose agar (PDA) medium, consisting of 200 g/L of potato, 30 g/L of NaCl, 15 g/L of agar, and 20 g/L of dextrose and incubated for three days at 28 °C with 60% humidity. Then, a single colony was transferred to a potato dextrose broth culture (comprising 200 g/L of potato, 20 g/L of peptone and 20 g/L of glucose,) and treated with different histone deacetylase inhibitors (niacinamide, SBHA and SAHA) for 10 days in a shaking flask at 28 °C. After fermentation, an examination of the fungal morphology was conducted on the three different media types, and (SMs) were extracted with ethyl acetate for further analysis.

### 4.2. Extraction and Analysis by HPLC and LC-MS

After 10 days of culture, the PDB medium was extracted 3 times by ethyl acetate. The resulting extract was then concentrated using a rotary evaporator operating at 120 rpm and 38 °C under vacuum. Subsequently, the dried extract was redissolved with 1 mL of methanol and centrifuged at 1200× *g* for 5 min. All measurements were performed with a Waters HPLC instrument comprising a 1525 pump and 2998 detector. A TC-C18 (2), 4.6 mm × 150 mm, 5 μm column (Agilent) was used to separate 20 μL of the sample via a linear gradient from (A) water with 0.2% acetic acid to (B) acetonitrile with 0.2% acetic acid at a flow rate of 1 mL/min. The gradient increased from 20% to 80% B in 30 min to end up with 10 min 100% B. Each sample had three separate biological duplicates. The LC flow rate was 0.6 mL/min before entering the LTQ Orbitrap XL mass spectrometer (Thermo Fisher Scientific, Hemel Hempstead, UK) with an electrospray interface (ESI). The spray voltage was set to 4.2 kV, the sheath gas pressure was set to 35 arb, and the auxiliary gas pressure was set to 10 arb. The heater temperature was set to 320 °C, and the capillary temperature was set to 300 °C. The mass spectra were acquired ranging from 120 to 1000 Da in the full-scan positive ESI mode, complemented by MS/MS scans of the three most intense ions from each full-scan at a collision energy of 35 V.

### 4.3. Structural Annotation of Compounds

The approach established by our group, which incorporates computational programs, MS-DIAL, SIRIUS and web-based tools, including MetaboAnalyst and GNPS [13], was utilized to analyze and determine the compounds in the extracts. In brief, the LC-MS/MS raw data were pre-processed using MS-DIAL [57], which included peak list alignment, adduct ion matching and minimum peak height threshold setting. The aligned dataset was submitted to MetaboAnalyst for PLS-DA and heatmap analysis. The structures of compounds of interest were identified at four levels. First, the structures were annotated using MS-DIAL linked MS/MS databases, taking into account the distinctive product ions and neutral losses at level 1. Second, the compounds that did not match in MSP were annotated on SIRIUS software (version 5.8.6) at level 2. SIRIUS could determine the molecular formulas, predict compounds’ molecular fingerprints using the ‘Predict FPs’ function and search the compounds using the ‘Search DBs’ function with CSI: FingerID in databases such as Natural Products, PubChem, PubMed, and Bio Database, etc. [58]. Third, some compounds that were still not recognized were assisted by GNPS at level 3. The global natural product social molecular network (GNPS) was utilized to investigate the relationship between the compounds’ structures. Finally, compounds that could not be identified by the above three methods were purified, isolated and analyzed in terms of NMR spectrum at level 4. The specific parameter settings of each level referred to previous articles.

### 4.4. Isolation and Purification of the SMs

The whole fermented PDB medium (5 L) was extracted three times with ethyl acetate (5 L) after 10 days of growth and then evaporated under reduced pressure to give a crude extract (20 g). *N*-hexane was then used to extract the crude extract. The extract powder was fractionated using Ez purification III’s silica gel column using different proportions of chloroform–methanol mixed solvents from 100:0 to 50:50 to yield 6 fractions (Frs.1-6) based on TLC. Frs. 3 separated using Sephadex LH-20 CC (MeOH) to give five subfractions (Frs.3.1–3.5). Frs.3.2 and Frs.3.4 were purified via preparative HPLC with 80% and 65% acetonitrile to obtain compounds A11 (10 mg), S1 (3 mg) and S2 (4 mg). Compounds A2 (21 mg), A3 (32 mg) and A9 (8 mg) were purified from Frs.4 using the preparative HPLC with an isocratic elution of acetonitrile-H_2_O (40% acetonitrile) and then further purified with semipreparative HPLC (60% acetonitrile). Frs.5 was purified via DAISO ODS column eluting with an acetonitrile-H_2_O gradient (from 3:7 to 10:0) at a flow rate of 20 mL/min to yield 3 subfractions (Frs.5.1–5.3). Frs.5.2 was further purified via preparative HPLC with a 60% acetonitrile isocratic elution to yield compound A8 (16 mg). Frs.2 was purified via MPLC twice to yield compounds A4 (16 mg) and A13 (15 mg).

### 4.5. Computational Details

Stochastic conformational searches for compound A11 were performed using molecular operating environment (MOE) software and putative diastereomers having relative energies within 2 kcal/mol. The conformations were further optimized at the B3LYP/6-31G (d) level in MeOH using Gaussian 09 and calculated by GaussView 5.0. Then, the optimized conformers were subjected to the time-dependent density functional theory (TDDFT) at B3LYP/6-31G (d, p) level in MeOH. The Gaussian function with bandwidth σ = 0.30 eV was used to simulate the ECD spectra. Finally, by measuring the Boltzmann distribution rate of each geometric conformation, the ECD spectra of compound A11 were determined [59].

### 4.6. Bioactivity Evaluation

The diabetes-related PTPs (PTP1B, TCPTP, SHP1 and CD45) inhibitory activity of the tested compounds was measured as previously described [60]. The compounds were dissolved in DMSO and incubated at 37 °C with 2.5 μg domain protein, 20 mM 3-(N-Morpholino) propanesulfonic (MOPS), 9 mM p-nitrophenyl phosphate (p-NPP), 50 mM NaCl and 50 mM DTT, pH 7.2. The reaction was performed in a 96-well plate (DMSO as blank control). The reaction was started by adding the assay buffer (50 mM citrate (pH 6.0), 0.1 M NaCl, 1 mM EDTA, and 1 mM dithiothreitol) with a final volume of 150 μL and incubated at 37 °C for 30 min and 10 M NaOH was added to terminate the reaction. The inhibition of PTPs by tested compounds was assessed by measuring the degradation of p-NPP at a wavelength of 405 nm.

### 4.7. Molecular Docking

The binding sites between SHP1 and the compounds were predicted using the Schrodinger Maestro 10.2 software package. The 3D structures of A3 and A13 were portrayed using Chem 3D. The crystal structure of SHP1 (PDB: 4GRY) was downloaded from the Protein Data Bank (www.rcsb.org) (accessed on 2 November 2023). The polar hydrogen atoms were added to SHP1 first, and water was removed to obtain a stable receptor. Considering protein structure, the three best-ranked cavities with site scores of >0.9 and volumes of >60 were chosen as the predicted sites. The docking grid (20 × 20 × 20 Å^3^) was constructed based on the binding sites that were chosen. A3 and A13 were docked with SHP1 separately to predict the binding site. The binding location with the lowest docking energy for the conformation was chosen. Parameters, including site score and glide score (kcal/mol), were obtained based on the docking results [61].

### 4.8. Statistical Analysis

All experiment data were repeated in triplicate and presented as means ± standard deviations (*n* = 3). Statistical analyses were established via SPSS 17.0 using one-way analysis of variance (ANOVA) followed by the Fisher LSD test, with a *p*-value of less than 0.05 taken as significant.

## 5. Conclusions

During studies to identify new PTP inhibitors from fungi using the CER strategy, significant changes in both growth and metabolite profiles were observed in the culture of *A. sydowii* DL1045 after adding a histone deacetylase inhibitor, subylanilide hydroxamic acid (SAHA). The metabolites analysis with an integrated metabolomics strategy established by our group found that 20 newly biosynthesized metabolites (10% of the total SMs) were detected only in the SAHA broth, and one of the newly biosynthesized metabolites (sydowimide A) was elucidated as a novel compound. This compound showed various inhibitory activities against PTPs, including SHP1, TCPTP and C45. The biotransformation of SAHA by *A. sydowii* DL1045 was also confirmed. Our study revealed that CER effectively altered the secondary metabolic profile of fungi, enhancing the diversity of bioactive metabolites, and the PTPs’ inhibitors obtained in this study have the potential to be developed as drug candidates associated with PTP inhibition, although further in vivo experiments are still needed.

## Figures and Tables

**Table 1:** The 20 newly biosynthesized features of *A. sydowii* 1045 by chemical epigenetic regulation (CER).

No.	tR [min]	Observed [*m*/*z*]	Adduction	Molecular Formula	Error (ppm)	Identification	Originally Isolated from	First Report in *A. sydowii*	Identification Method (Level)
A1	24.472	217.1585	[M+H]^+^	C_15_H_20_O	−0.929	(−)-1,2-dehydro-α-cyperone	*Tussilago farfara* [22]	Y	2
A2	26.640	229.0876	[M+H]^+^	C_14_H_12_O_3_	−0.630	3,7-dihydroxy-1,9-dimethyldibenzofuran	*A. sydowii* [23]	N	2, 4
A3	22.818	231.1015	[M+H]^+^	C_14_H_14_O_3_	−0.040	diorcinol	*A. sydowii* [5]	N	2, 4
A4	24.341	235.1692	[M+H]^+^	C_15_H_22_O_2_	−1.032	aspergillusene A	*A. sydowii* [5]	N	2, 4
A5	20.616	239.1282	[M+H]^+^	C_13_H_18_O_4_	0.766	1-(3-ethyl-2,4-dihydroxy-6-methoxyphenyl) butan-1-one deoxyphomalone	*Alternaria* sp. [24]	Y	1
A6	21.301	253.1438	[M+H]^+^	C_13_H_16_O_5_	−2.915	methyl trimethoxycinnamate	*Staphylococcus aureus* [25]	Y	2
A7	7.868	256.1177	[M+H]^+^	C_12_H_17_NO_5_	−2.958	radicamine A	*Codonopsis pilosula* [26]	Y	2
A8	25.210	267.1586	[M+H]^+^	C_15_H_22_O_4_	−3.758	sydonic acid	*A. sydowii* [23]	N	2
A9	23.068	275.0912	[M+H]^+^	C_15_H_14_O_5_	−2.866	lecanorin	*Parmotrema tinctorum* [27]	Y	2, 4
A10	23.259	286.1441	[M+Na]^+^	C_17_H_19_NO_3_	0.872	piperine	*Staphylococcus aureus* [25]	Y	1
A11	12.592	289.1183	[M+H]^+^	C_15_H_16_N_2_O_4_	−1.874	sydowimide A	*A. sydowii*	Y	4
A12	15.624	299.0903	[M+H]^+^	C_17_H_14_O_5_	0.329	emodin-1,6-dimethyl ether	*Penicillium* sp. [28]	Y	2
A13	15.250	319.0812	[M+H]^+^	C_16_H_14_O_7_	−1.779	diorcinol acid	*A. sydowii* [21]	N	2
A14	28.042	319.0813	[M+H]^+^	C_16_H_14_O_7_	−1.778	lecanoric acid	*Pyricularia* [29]	Y	2
A15	28.391	328.1542	[M+H]^+^	C_19_H_21_NO_4_	−2.173	scoulerine	*Argemone* [30]	Y	1
A16	22.457	347.1251	[M+Na]^+^	C_20_H_20_O_4_	−2.219	bavachin	*Cullen corylifolium* [31]	Y	1
A17	22.909	393.2939	[M+H]^+^	C_24_H_40_O_4_	−0.654	deoxycholic acid	*Pseudomonas syringae* [32]	Y	1
A18	19.735	417.2390	[M+H]^+^	C_23_H_32_N_2_O_5_	−0.402	*N*-Acetyl-*N*-depropionyl-aspido-albinol	*Aspidosperma album* [33]	Y	2
A19	24.691	458.3265	[M+H]^+^	C_28_H_43_NO_4_	1.588	2-((2*E*,5*E*,7*E*,11*E*)-10-Hydroxy-3,5,7,9,11,13-hexamethyl-tetradeca-2,5,7,11-tetraenyl)-5,6-dimethoxy-3-methyl-pyridin-4-ol	*Streptomyces pactum* [34]	Y	2
A20	22.412	517.2373	[M+Na]^+^	C_25_H_34_O_10_	−0.306	rubranoside A	*Alnus hirsuta f. sibirica* [35]	Y	1

Y: reported in *A. sydowii* firstly; N: detected in other *A. sydowii* except for *A. sydowii* 1045.

**Table 2:** ^1^H (500 MHz, DMSO-*d*6) and ^13^C (250 MHz, DMSO-*d*6) NMR analysis of sydowimide A (A11a and A11b).

NO.	A11a		A11b	
	δC	δH (*J* in Hz)	δC	δH (*J* in Hz)
1				
2	180.05		179.70	
3	36.51	2.4 (1H, m)	36.13	2.4 (1H, m)
4	34.58	3.01 (2H, m)	34.67	3.01 (2H, m)
5	175.98		175.86	
6	95.62		95.68	
7	164.19		164.25	
8	140.41	8.08 (1H, d, 10)	140.41	8.08 (1H, d, 10)
NH		9.22 (d, *J* = 13.5 Hz, 1H)		9.17 (d, *J* = 13.6 Hz, 1H)
10	139.59		139.67	
11	116.01	7.16 (1H, m)	116.27	7.16 (1H, m)
12	129.61	7.33 (1H, m)	129.61	7.33 (1H, m)
13	122.80	7.03 (1H, m)	122.90	7.03 (1H, m)
14	129.61	7.33 (1H, m)	129.61	7.33 (1H, m)
15	116.01	7.16 (1H, m)	116.27	7.16 (1H, m)
16	16.64	1.25 (d, *J* = 6.8 Hz, 3H)	15.25	1.33 (d, *J* = 6.7 Hz, 3H)
17	51.24	3.62 (3H, s)	51.24	3.62 (3H, s)

**Table 3:** Activities of newly induced compounds against protein tyrosine phosphatases (PTPs).

		IC50 (μM)		
	SHP1	TCPTP	PTP1B	CD45
A3	0.96 ± 0.02	>20	>20	4.03 ± 0.13
A4	8.69 ± 0.26	>20	9.65 ± 0.73	>20
A9	6.01 ± 0.18	>20	7.60 ± 0.21	7.40 ± 0.08
A11	1.50 ± 0.09	2.40 ± 0.65	>20	18.83 ± 0.04
A13	2.16 ± 0.23	>20	>20	4.46 ± 0.16

**Table 4:** Binding Glide Scores of A3 and A13 on the Src homology region 2 domain-containing phosphatase-1 (SHP1).

	Site	Site Score	Docking Glide Score (kcal/mol)	
			A3	A13
SHP1	2	0.927	−5.622	−4.588
	1	0.903	−4.356	−4.135
	3	0.706	−3.002	−4.832

## Data Availability

The data presented in this study are available in Supplementary Material.

## Supplementary Materials

The following supporting information can be downloaded at: https://www.mdpi.com/article/10.3390/molecules29030670/s1, Figure S1: The weight of metabolite ethyl acetate extract in SAHA group and control after 10 days; Figure S2: The effects of SAHA concentrations on the metabolites after 10 days; Figure S3: Positive and negative HRESIMS spectrum of sydowimide A (**A11**); Figure S4: IR spectrum of sydowimide A (**A11**); Figure S5: ^1^H NMR (500 MHz, DMSO-*d*_6_) spectrum of sydowimide A (**A11**); Figure S6: ^13^C NMR (250 MHz, DMSO-d6) spectra of sydowimide A (**A11**); Figure S7: H-H COSY spectrum of sydowimide A (**A11**); Figure S8: HMQC spectrum of sydowimide A (**A11**); Figure S9: HMBC spectrum of sydowimide A (**A11**); Figure S10: Optimized conformers (1%) of sydowimide A (**A11**) at the B3LYP/6-31+G (d) level with PCM model in MeOH; Figure S11: Total of 20 features induced only after the CER were identified by the combination of the computational approach; Figure S12: The content of SAHA before and after fermentation; Figure S13: Representation of the binding mode of compounds A11a (a,c) and A11b (b,d) in the active site and the inactive site of SHP1; Table S1: The characteristics of increased and decreased in abundance following SAHA treatment, respectively; Table S2: Important Thermodynamic Parameters and Conformational Analysis of sydowimide A (**A11**); Table S3: Optimized Cartesian Coordinate of compound sydowimide A (**A11**) at B3LYP/6-31G (d, p) level in MeOH Using the PCM Model; Table S4: Activities of newly induced compounds against nematode; Table S5: Binding Glide Scores of A11a and A11b on the SHP1; Table S6: Physicochemical descriptors as well as to predict ADME parameters, pharmacokinetic properties by SwissADME of detected compounds.
