# Supplementary material for: Chemical Epigenetic Regulation Secondary Metabolites Derived from Aspergillus sydowii DL1045 with Inhibitory Activities for Protein Tyrosine Phosphatases
Source: Molecules. 2024 Jan 31;29(3):670. doi: 10.3390/molecules29030670 (PMC10856041; doi:10.3390/molecules29030670)
Supplement: Supplementary file 1 [file molecules-29-00670-s001.zip › molecules-2783361-SM.pdf]

Chemical Epigenetic Regulation Secondary Metabolites Derived from *Aspergillus sydowii* DL1045 with Inhibitory Activities for Protein Tyrosine Phosphatases

Xuan Shi [1], Xia Li [1], Xiaoshi He [1], Danyang Zhang [1], Chunshan Quan [2], Zhilong Xiu [1] and Yuesheng Dong [1,*]

1 School of Bioengineering, Dalian University of Technology, Dalian 116024, China
2 College of Life Science, Dalian Minzu University, Dalian 116600, China
* Correspondence: yshdong@dlut.edu.cn.

Supplementary Information

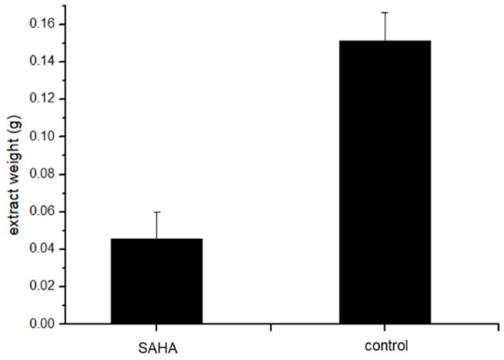

Figure S1. The weight of metabolite ethyl acetate extract in SAHA group and control after 10 days.

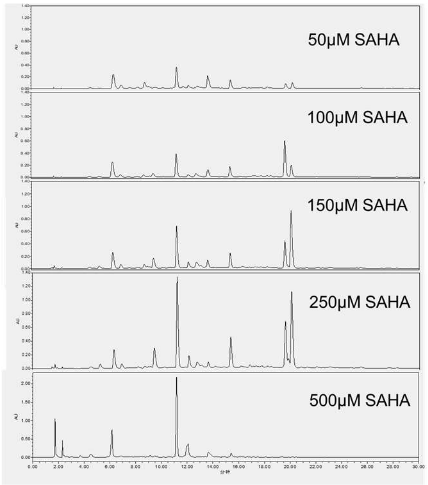

Figure S2. The effects of SAHA concentrations on the metabolites after 10 days.

Table S1 The characteristics of increased and decreased in abundance following SAHA treatment, respectively.

	Increased			Decreased	
NO.	RT (min)	m/z	NO.	RT (min)	m/z
345	22.103	252.1897	94	13.983	179.1426
523	13.937	287.1359	151	13.923	197.1533
673	22.451	329.1016	419	16.311	259.0596
735	22.657	347.1132	652	21.543	321.1666
217	30.554	227.1464	745	16.224	356.1372
211	24.274	247.2303	767	22.169	365.2776
323	21.623	239.1654	784	14.177	375.1062
483	14.933	271.1407	805	15.749	386.2445
360	21.503	250.1442	853	19.72	409.2547
1073	16.14	481.1871	1065	13.555	479.2311
868	26.762	415.2116	1099	27.056	490.3685
104	11.745	181.1227	1293	19.012	583.313
70	7.222	169.0498	1298	19.249	601.3374
108	13.67	182.0815	1321	16.131	857.3807

236	14.808	222.1128	615	20.775	305.1726
522	18.122	282.1701	613	20.308	305.1726
175	3.57	206.0451	1244	12.275	541.3399
359	19.571	250.1442	1296	13.147	599.3814
460	11.393	267.1225	169	15.758	203.143
458	12.581	266.175	623	15.543	309.13
1069	13.584	481.1871	790	29.649	377.2286
99	14.268	181.1218	1303	13.403	643.4067
936	18.696	439.1736	1054	24.317	477.0823
1310	16.225	667.3031	87	13.339	177.1271
79	6.957	171.0647	1301	13.276	621.3911
525	14.312	283.118	1199	12.117	519.3232
443	11.221	265.1542	588	11.837	301.0697
777	26.796	369.2634	424	20.223	261.1484
161	11.123	201.1118	578	11.672	299.0529
572	9.928	296.1493	188	8.047	211.0605
472	4.383	268.1033	93	13.544	179.1426
			1320	16.169	835.4006
			659	29.649	323.2225
			559	16.764	291.0859
			219	8.94	218.1035
			716	17.468	345.0602
			1214	18.559	525.3078
			519	21.486	281.1754
			582	21.514	299.1857
			846	21.399	406.3517
			444	10.737	265.1542
			873	28.803	415.2691
			268	7.005	231.0864

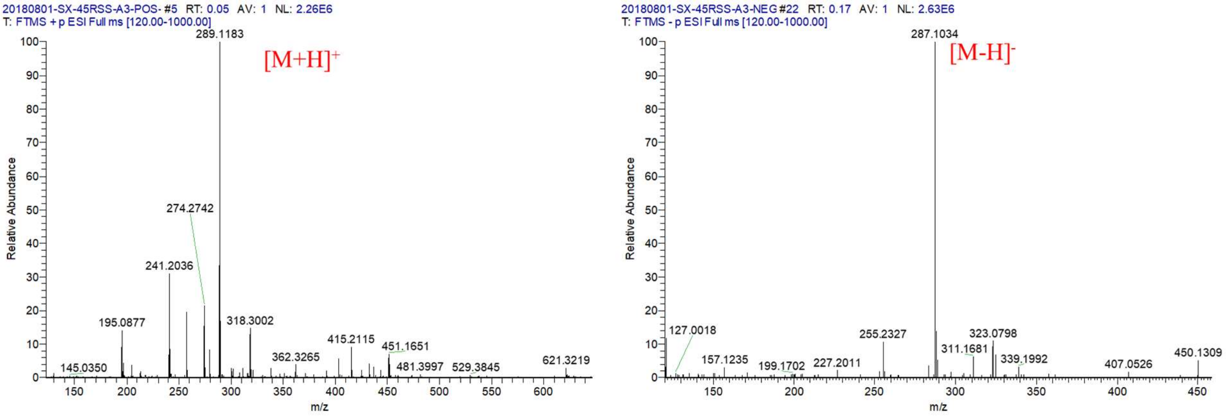

Figure S3 Positive and negative HRESIMS spectrum of sydowimide A **(A11)**.

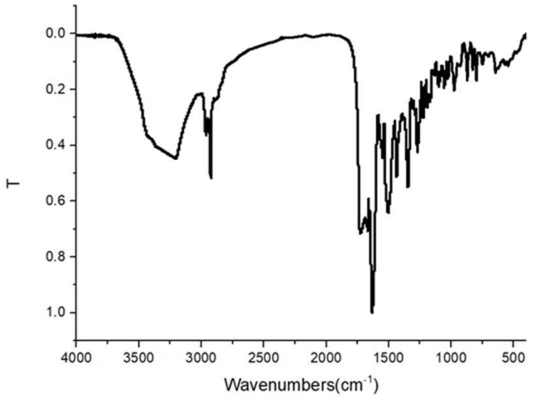

Figure S4 IR spectrum of sydowimide A **(A11)**.

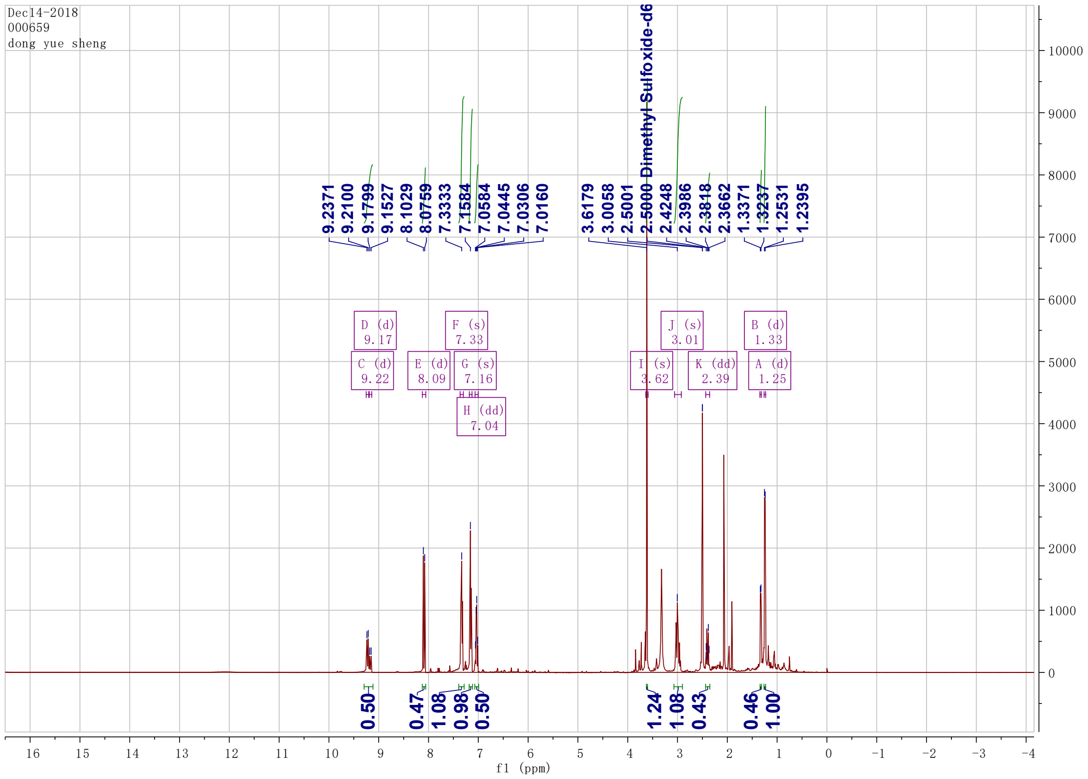

Figure S5 ^{1}H NMR (500 MHz, DMSO-d_6) spectrum of sydowimide A (**A11**).

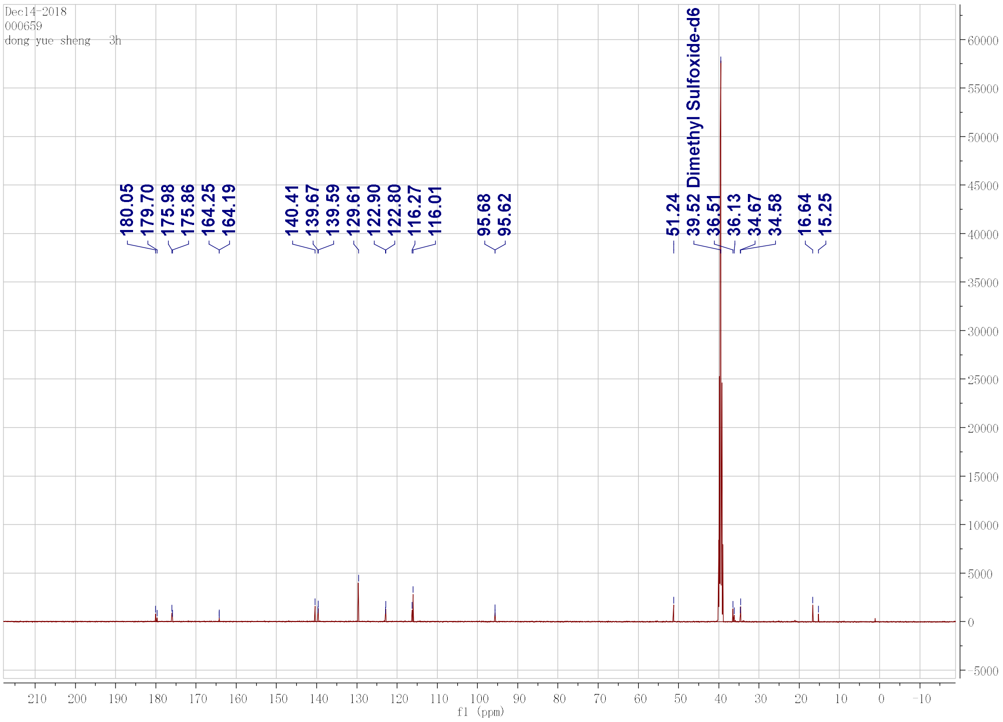

Figure S6 ^{13}C NMR (250 MHz, DMSO-d_6) spectra of sydowimide A (**A11**).

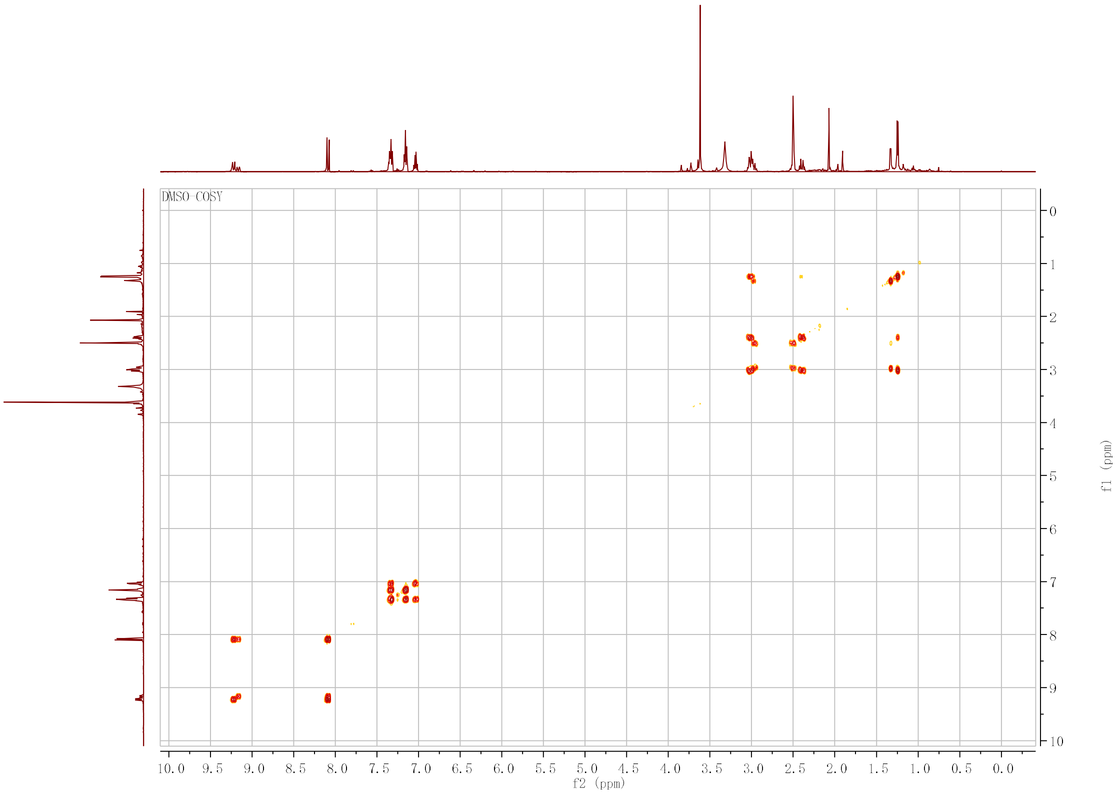

Figure S7 H-H COSY spectrum of sydowimide A (**A11**).

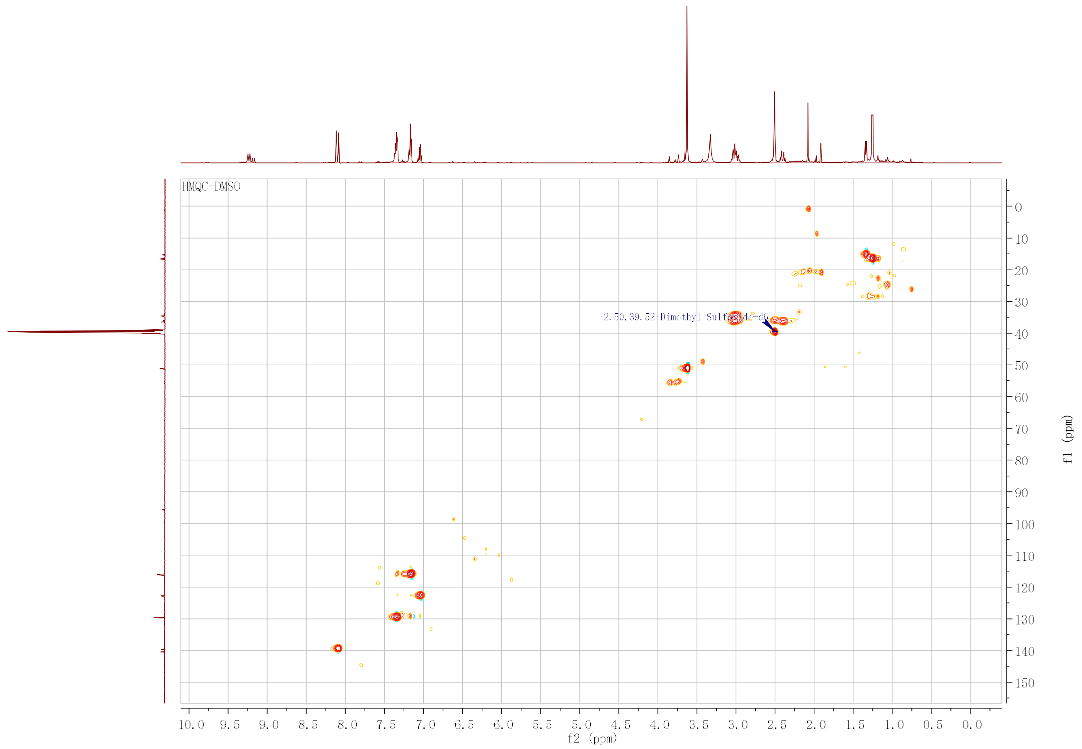

Figure S8 HMQC spectrum of sydowimide A (**A11**).

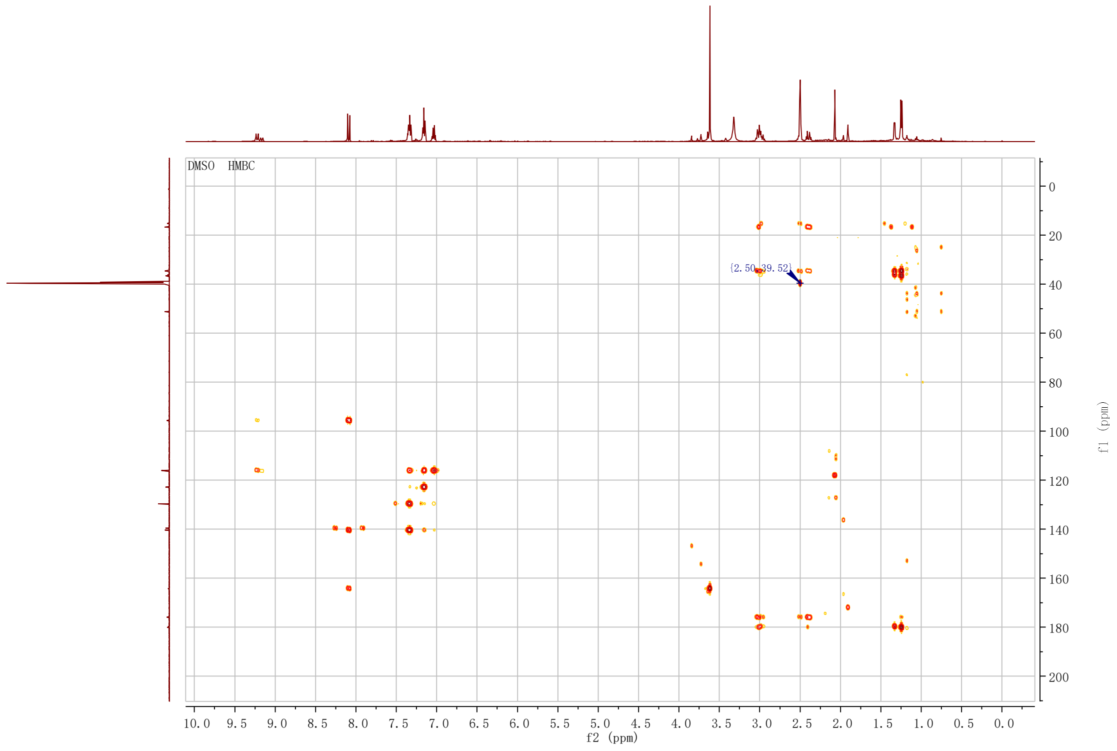

Figure S9 HMBC spectrum of sydowimide A (**A11**).

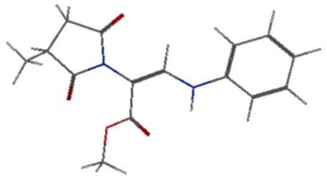

A11-1

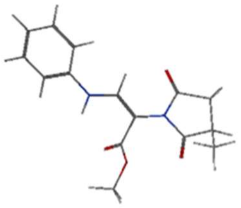

A11-2

Figure S10 Optimized conformers (≥ 1%) of sydowimide A (**A11**) at the B3LYP/6-31+G (d) level with PCM model in MeOH.

Table S2. Important Thermodynamic Parameters and Conformational Analysis of sydowimide A (**A11**).

Conformations	E+ZPE	G	P
A11-1	-934.969575	-934.988547	88.40%
A11-2	-934.969575	-934.969575	11.60%

E+ZPE: total energy with zero point energy; G: Gibbs free energy; P: conformational distributions calculated from relative Gibbs free energy.

Table S3. Optimized Cartesian Coordinate of compound sydowimide A (**A11**) at B3LYP/6-31G (d, p) level in MeOH Using the PCM Model

No.	Atom	A11-1			A11-2		
		X	Y	Z	X	Y	Z
1	C	3.48927	-1.836175	-0.448678	-3.489249	-1.836203	-0.448649
2	C	2.327212	-0.974381	-0.937563	-2.327184	-0.974428	-0.937549
3	C	3.525053	-1.593984	1.071801	-3.525079	-1.593925	1.071814
4	C	-5.861346	-1.002806	-0.100224	5.861338	-1.00282	-0.100218
5	C	4.788267	-1.530869	-1.201271	-4.788229	-1.530954	-1.201296
6	C	-4.808635	-1.869073	0.203436	5.582963	0.338967	-0.371933
7	C	2.267896	-0.803897	1.393106	-2.267928	-0.803829	1.393112
8	C	-5.58297	0.338991	-0.371885	4.808628	-1.869068	0.203502
9	C	-4.271129	0.807186	-0.348278	3.491336	-1.411905	0.237458
10	C	0.498134	0.367853	0.090751	-0.498135	0.367861	0.090753
11	C	2.24237	3.590832	0.017888	-2.24237	3.590841	0.017823

12	C	-3.491339	-1.411919	0.237384	4.271126	0.807169	-0.34832
13	C	-3.21431	-0.066379	-0.047152	3.214307	-0.066376	-0.047133
14	C	0.690231	1.808817	0.025823	-0.690233	1.808825	0.025802
15	C	-0.749208	-0.205417	0.060562	0.749207	-0.205409	0.060585
16	H	5.582973	-2.211445	-0.880321	-5.582942	-2.211506	-0.88031
17	H	-6.883883	-1.367445	-0.120581	6.883872	-1.367465	-0.120581
18	H	5.119597	-0.503797	-1.011463	-5.119565	-0.503867	-1.011577
19	H	3.55098	-2.509968	1.668002	-3.551037	-2.509872	1.66807
20	H	-6.388775	1.028563	-0.607082	5.00928	-2.9134	0.425723
21	H	-0.813437	-1.286687	0.11117	0.813439	-1.286677	0.111212
22	H	4.3892	-0.988006	1.369645	-4.389231	-0.987924	1.369595
23	H	-4.059142	1.850543	-0.567208	2.697447	-2.103722	0.497546
24	H	-2.69745	-2.103754	0.497421	4.059139	1.850518	-0.567293
25	H	-1.80505	1.479542	-0.114618	1.805051	1.479548	-0.11462
26	H	4.642736	-1.651487	-2.278639	-4.642675	-1.651657	-2.278652
27	H	1.864268	4.009979	-0.917749	-1.864272	4.00997	-0.917824
28	H	1.770553	4.101146	0.861138	-3.326108	3.691248	0.069832
29	H	3.198407	-2.875032	-0.652876	-3.198373	-2.875069	-0.652783
30	H	3.326108	3.691239	0.069895	-1.770549	4.101168	0.861062
31	H	-5.009288	-2.913414	0.425613	6.388767	1.028523	-0.607177
32	N	-1.910939	0.467449	-0.036523	1.91094	0.467457	-0.036496
33	N	1.638334	-0.484239	0.180903	-1.638336	-0.484228	0.180911
34	O	1.986766	2.17542	0.077067	-1.986767	2.175428	0.077025
35	O	-0.229945	2.62533	-0.066743	0.229945	2.625336	-0.066768
36	O	1.856613	-0.472662	2.486941	-1.856671	-0.472545	2.486943
37	O	2.014499	-0.746227	-2.089415	-2.014441	-0.746332	-2.089404

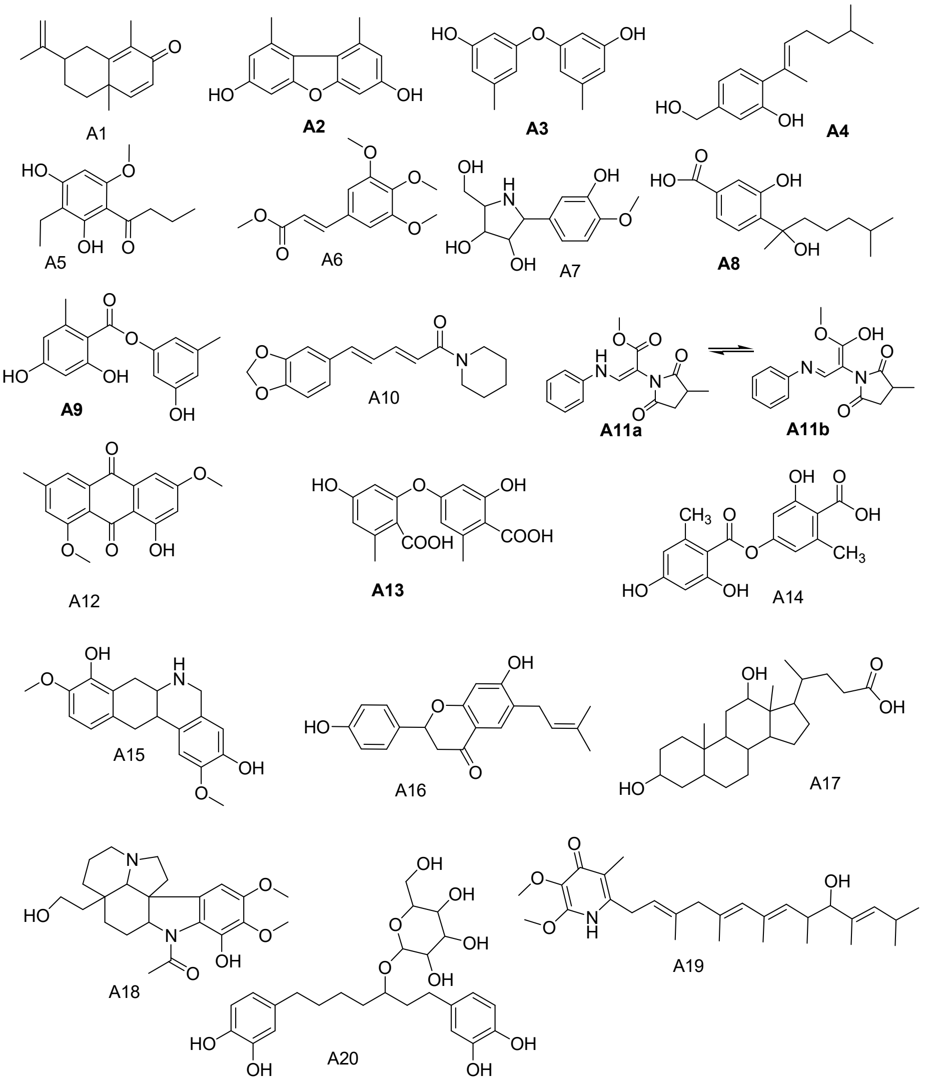

Figure S11 Total of 20 features induced only after the CER were identified by the combination of the computational approach.

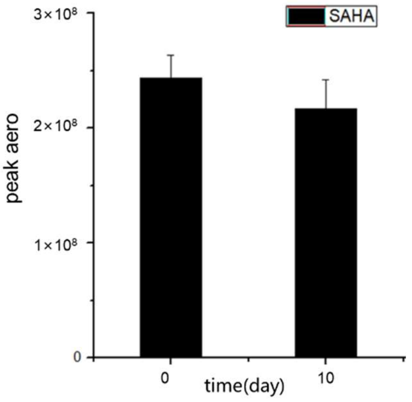

Figure S12 The content of SAHA before and after fermentation.

Table S4. Activities of newly induced compounds against nematode.

	IC50(μM)
	nematode
A3	50
A4	>100
A9	>100
A11	>100
A13	>100

Table S5. Binding Glide Scores of A11a and A11b on the SHP1.

	Docking glide score (Kcal/mol)			
	site	site score	A11a	A11b
SHP1	2（active site）	0.927	-4.054	-4.815
	1	0.903	-3.703	-4.456
	3	0.706	-3.600	-5.586

a.

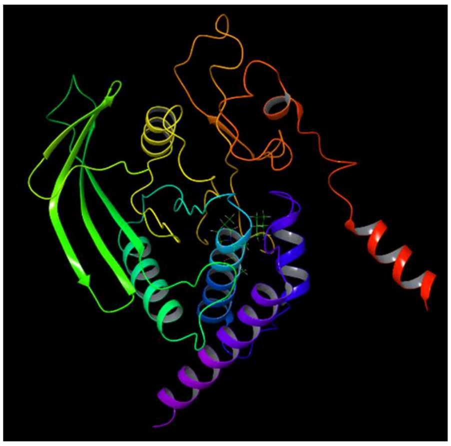

b.

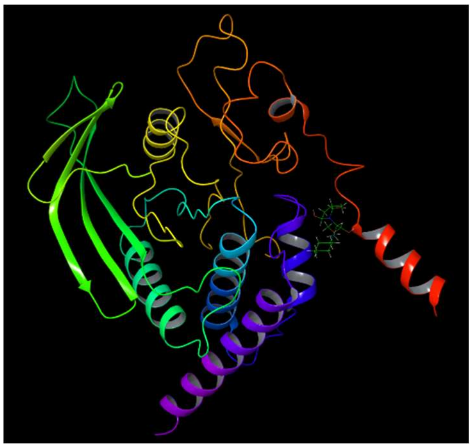

c.

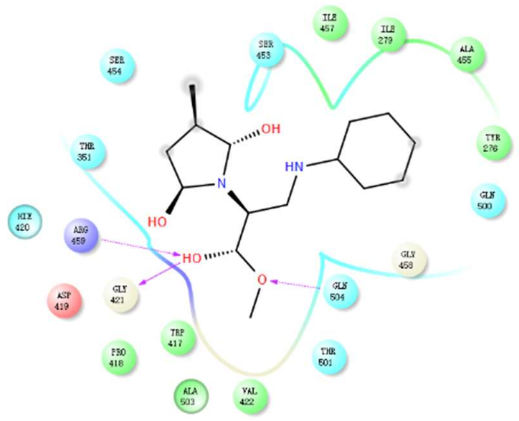

d.

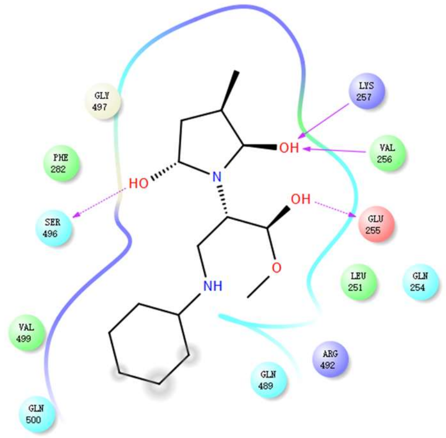

Figure S13. Representation of the binding mode of compounds A11a (a, c) and A11b (b, d) in the active site and the inactive site of SHP1.

Table S6. Physicochemical descriptors as well as to predict ADME parameters, pharmacokinetic properties by SwissADME of detected compounds.

		**Lipophilicity characteristics**						**Water solubility characteristics**				**Pharmacokinetics parameters and bioavailability**			**Drug-likeness rules score**				
Molecule	MW	iLOGP	XLOGP3	WLOGP	MLOGP	Silicos -IT Log P	Consensus Log P	Log S	Solubility (mg/ml)	Solubility (mol/l)	Class	GI absorption	BBB permeant	Bioavailability Score	Lipinski	Ghose	Veber	Egan	Muegge
A3	230.26	2.35	3.47	3.51	2.6	3.03	2.99	-4.2	1.47E-02	6.38E-05	Moderately soluble	High	Yes	0.55	0	0	0	0	0
A4	234.33	3.26	4.08	3.57	3.11	3.76	3.56	-4.63	5.43E-03	2.32E-05	Moderately soluble	High	Yes	0.55	0	0	0	0	0
A9	274.27	2.11	3.55	2.64	2.18	2.46	2.59	-5.06	2.38E-03	8.67E-06	Moderately soluble	High	No	0.55	0	0	0	0	0
A11	288.3	2.43	2.01	0.94	0.72	1.13	1.44	-3.23	1.71E-01	5.93E-04	Soluble	High	No	0.55	0	0	0	0	0
A13	318.28	0.2	3.07	2.9	1.55	1.92	1.93	-5.35	1.43E-03	4.50E-06	Moderately soluble	High	No	0.56	0	0	0	0	0

Compounds information

sydowimide A **(A11)**: yellow oil; HRESIMS m/z 289.1185 $[M+H]^+$ and 287.1034$[M-H]^-$ (calcd for $C_{15}H_{16}O_4N_2$, 288.1110); ^{1}H and ^{13}C NMR as shown in Table 2 and S6-S7;

3,7-dihydroxy-1,9-dimethyldibenzofuran **(A2)**: yellow oil; HRESI(+)MS m/z 367.1376 $[M+H]^+$ (calcd for $C_{14}H_{12}O_3$, 228.0786).^{1}H-NMR (500 MHz, DMSO-*d6*):1.24 (3H, s), 1.57(3H, s), 1.96 (3H, s), 2.14 (3H, s), 3.05 (3H, s), 4.27(1H, s), 4.64(1H, s), 6.39(1H, s), 9.52(1H, s), 10.26(1H, s), 12.33(1H, s). ^{13}C-NMR (125.76 MHz, DMSO-*d6*):7.61, 9.56, 13.79, 19.59, 50.02, 70.18, 80.43, 83.31, 107.09, 110.95, 114.20, 119.44, 132.91, 154.78, 156.43, 169.11, 171.96, 192.7.

diorcinol (**A3**): yellow oil; HRESIMS m/z 231.1015 $[M+H]^+$ (calcd for $C_{14}H_{14}O_3$, 230.0943), ^{1}H-NMR (500 MHz, CD_3OD): 6.44 (1H, s), 6.31 (1H, s), 6.29 (1H, d), 6.24 (1H, t), 6.16(1H, d), 2.46 (3H,s), 2.22 (1H, s). ^{13}C-NMR (125.76 MHz, CD3OD): 21.50, 24.17, 103.60, 105.75, 108.37, 113.12, 113.30, 113.56, 142.06, 145.30, 157.34, 159.84, 163.68, 166.33, 174.67.

aspergillusene A (**A4**): yellow oil; HRESIMS m/z 235.1692 $[M+H]^+$ (calcd for $C_{15}H_{22}O_2$, 234.1620), ^{1}H-NMR (500 MHz, CD_3OD): 6.96 (1H, d), 6.74 (1H, m), 6.74 (1H, m), 5.39 (1H, t), 4.49 (2H, s), 2.15 (2H, m), 1.94(3H, s), 1.62 (1H, m), 1.31 (2H, dt), 0.93 (3H, d), 0.93 (3H, d). ^{13}C-NMR (125.76 MHz, CD_3OD): 17.22, 23.00, 27.21, 28.92, 39.97, 65.03, 114.96, 119.05, 130.42, 130.79, 133.26, 135.53, 142.24, 155.24.

Sydonic acid (**A8**): Colorless needles; ESI-MS m/z: 267.1586 $[M+H]^+$ (calcd for $C_{15}H_{22}O_4$, 266.1518); ^{1}H-NMR (500 MHz, CD_3OD): 0.80 (3H, s), 0.82 (3H, s), 1.13 (2H, m), 1.29 (1H, m), 1.34 (1H, m), 1.47 (1H, m), 1.59 (3H, s), 1.77 (1H, ddd), 1.94 (1H, ddd), 7.25 (1H, d,7.9), 7.37 (1H, d, 7.6), 7.44 (1H, dd). ^{13}C-NMR (125.76 MHz, CD_3OD): 22.83, 22.89, 22.96, 28.86, 28.95, 40.42, 43.68, 78.0, 118.67, 121.52, 127.74, 131.64, 137.91, 156.96, 169.88.

lecanorin(**A9**): yellow oil; HRESIMS m/z 275.0912 $[M+H]^+$ (calcd for $C_{15}H_{14}O_5$, 274.0841), ^{1}H-NMR (500 MHz, CD_3OD): 6.44 (1H, s), 6.31 (1H, s), 6.29 (1H, d), 6.24 (1H, t), 6.16(1H, d), 2.46 (3H,s), 2.22 (1H, s). ^{13}C-NMR (125.76 MHz, CD3OD): 21.50, 24.17, 103.60, 105.75, 108.37, 113.12, 113.30, 113.56, 142.06, 145.30, 157.34, 159.84, 163.68, 166.33, 174.67.

diorcinolic acid(**A13**): yellow oil; HRESIMS m/z 319.0812 $[M+H]^+$ (calcd for $C_{16}H_{14}O_7$, 318.0740), ^{1}H-NMR (500 MHz, CD_3OD): 6.46 (1H, d), 6.25 (1H, d), 6.20 (1H, d), 6.14 (1H, d), 3.26 (3H,s), 2.42(3H, s), 2.26(3H, s). ^{13}C-NMR (125.76 MHz, CD_3OD):20.2, 24.09, 103.81, 106.42 ,108.97, 113.25, 114.89, 120.13, 140.49, 145.2, 155.04, 160.77, 163.25, 166.13, 170.84, 174.67.

suberanilic acid(**S2**): light yellow powder; HRESIMS m/z 250.144 $[M+H]^+$ (calcd for $C_{14}H_{19}NO_3$, 249.1365), ^{1}H-NMR (500 MHz, CD_3OD): 7.53 (2H, d), 7.29 (2H, t), 7.07 (1H, t), 2.36 (2H, t), 2.28 (2H,t), 1.71(2H, dd), 1.63(2H, m), 1.40(4H, m). ^{13}C-NMR (150.81 MHz, CD_3OD):26.10, 26.78, 29.98, 30.01, 35.28, 37.9, 121.26, 125.09, 129.74, 139.89, 174.63, 178.17.

3-hydroxybenzyl 8-oxo-8-(phenylamino)octanoate (**S3**): light yellow powder; HRESIMS m/z 249.160 $[M+H]^+$ (calcd for $C_{14}H_{20}N_2O_2$, 248.1525), ^{1}H-NMR (500 MHz, CD_3OD): 7.54 (2H, d), 7.30 (2H, t), 7.08 (1H, t), 2.37 (2H, t), 2.20 (2H, m), 1.71(2H, m), 1.64(2H, m), 1.41(4H, m). ^{13}C-NMR (150.81 MHz, CD_3OD): 26.73, 26.76, 29.98, 36.42, 37.88, 121.25, 125.10, 129.75, 139.90, 174.52, 179.22.
